# The efficacy of 'Radio guided Occult Lesion Localization' (ROLL) versus 'Wire-guided Localization' (WGL) in breast conserving surgery for non-palpable breast cancer: A randomized clinical trial – ROLL study

**DOI:** 10.1186/1471-2482-8-9

**Published:** 2008-05-21

**Authors:** Stijn van Esser, Monique GG Hobbelink, Petra HM Peeters, Erik Buskens, Iris M van der Ploeg, Willem PTHM Mali, Inne H M Borel Rinkes, Richard van Hillegersberg

**Affiliations:** 1 Department of Surgical Oncology, University Medical Center Utrecht, Heidelberglaan 100, G04.228 3584 CX Utrecht, The Netherlands; 2 Department of Nuclear Medicine, University medical Center Utrecht, Heidelberglaan 100, E01.132 3584 CX Utrecht, The Netherlands; 3 Department of Radiology, University Medical Center Utrecht, Heidelberglaan 100, E01.132 3584 CX Utrecht, The Netherlands; 4 Clinical epidemiology, Julius Center for Health Sciences and Primary Care, University Medical Center Utrecht, Heidelberglaan 100, STR 6.131 3584 CX Utrecht, The Netherlands; 5 Department of Epidemiology, University Medical Center Groningen, University of Groningen, PO box 30.001, E3.018, 9700 RB, Groningen, The Netherlands

## Abstract

**Background:**

With the increasing number of non palpable breast carcinomas, the need of a good and reliable localization method increases. Currently the wire guided localization (WGL) is the standard of care in most countries. Radio guided occult lesion localization (ROLL) is a new technique that may improve the oncological outcome, cost effectiveness, patient comfort and cosmetic outcome. However, the studies published hitherto are of poor quality providing less than convincing evidence to change the current standard of care.

The aim of this study is to compare the ROLL technique with the standard of care (WGL) regarding the percentage of tumour free margins, cost effectiveness, patient comfort and cosmetic outcome.

**Methods/design:**

The ROLL trial is a multi center randomized clinical trial. Over a period of 2–3 years 316 patients will be randomized between the ROLL and the WGL technique. With this number, the expected 15% difference in tumour free margins can be detected with a power of 80%. Other endpoints include cosmetic outcome, cost effectiveness, patient (dis)comfort, degree of difficulty of the procedures and the success rate of the sentinel node procedure.

The rationale, study design and planned analyses are described.

**Trial Registration:**

(, study protocol number NCT00539474)

## Background

The early detection of breast malignancies decreases the mortality and morbidity of breast cancer patients. These early-detected tumours are generally small and non-palpable.

Wire Guided Localization (WGL), is currently the most commonly used localization method for non-palpable breast lesions. This technique uses a wire to localize the lesion to be excised. The wire can be inserted under stereotactic or ultrasonographic guidance. WGL has several known disadvantages: the radiologically guided wire placement is technically difficult, particularly in dense breast tissue (the wire can displace and reposition is often restricted because of the hook fixed in the tissue). Surgical excision of a lesion with clear histological margins following wire localization is demanding as well. Finally, patients experience the inserted wire as painful and uncomfortable and there is a small risk of a pneumothorax [1-3].

The Radio Occult Lesion Localization (ROLL) introduced in 1998, is a new technique to localize the non-palpable breast tumour[4]. The ROLL technique utilizes the intratumourally injected radiofarmaceutical that is used for lymphatic mapping and sentinel node biopsy (SNB). In the same surgical procedure, this tracer can be used to localize the primary tumour guided by the gamma probe.

Previous small non randomized trials, comparing WGL and ROLL have found the ROLL technique to be simpler and faster to perform, potentially resulting in fewer costs associated with the use of ultrasound, operation rooms and hospital stay [5].

## Methods/Design

### Design

A multicenter, prospective randomized clinical trial. Eligible patients will be randomized for either radio guided occult lesion localization (ROLL) or wire guided localization (WGL).

### Subjects

Three hundred and sixteen patients will be recruited in 2 years in a University Medical Center and medium sized to large hospitals in the Netherlands.

All patients will have confirmed occult breast cancer (core needle biopsy proven) and need to be treated with a lumpectomy and sentinel node biopsy. Written informed consent will be obtained from all patients. This study has been approved by the ethical board of the university medical center Utrecht.

### Patient selection

All patients will be selected based on the in- and exclusion-criteria. The inclusion of patients will take place at the outpatient clinic in the participating hospitals. Patients will be informed about this trial by both written and oral explanation. Given the number of regular treatments of occult breast tumours in the collaborating hospitals (250–300 patients per year), the aim to finish the inclusion in 2 years is considered realistic.

Women ≥18 years with a non palpable breast carcinoma (cT1) that need to be treated with breast conserving surgery are asked to participate. Exclusion criteria are pregnancy, multifocal tumour growth, in situ ductal carcinoma only, lobular in situ carcinoma only and patients requiring breast amputation.

### Randomization

Randomization is stratified for hospital. In each hospital, randomization within strata is blocked with a fixed block size. Randomization is performed by an independent trial center. If a patient meets the inclusion criteria and has provided informed consent, the physician contacts the trial center by phone. The trial center will perform the randomization of the patients.

### Time schedule

Patient recruitment will take place between 2008 and 2010.

### ROLL procedure

**Figure 1:**
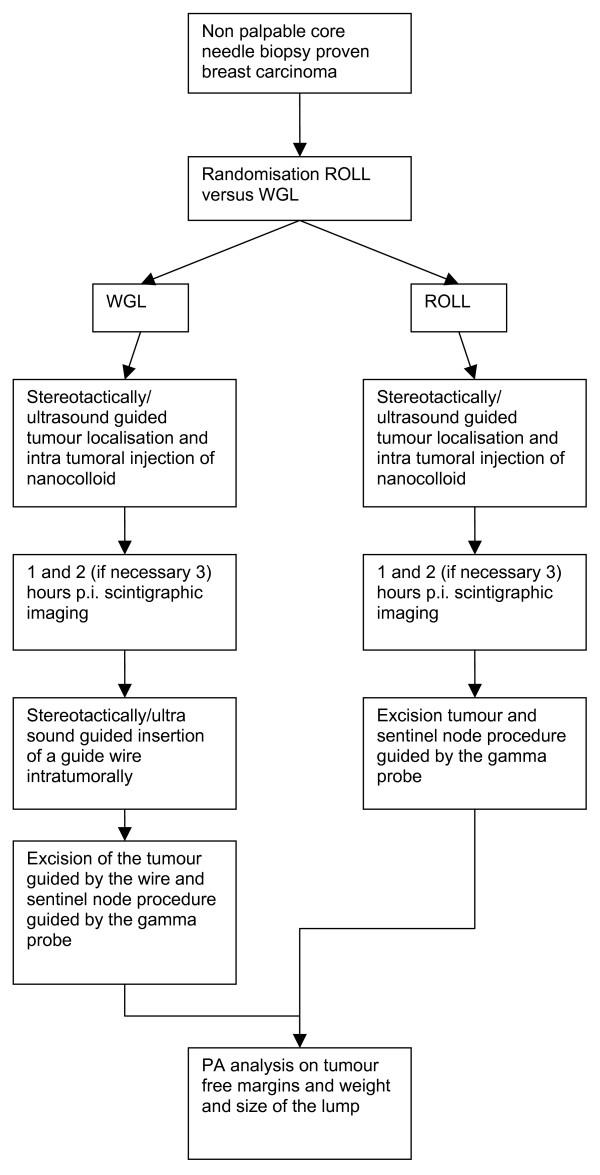
Flowchart.

Patients in the ROLL group will undergo intratumoural injection of the radiofarmaceutical under stereotactic or ultrasound guidance. After scintigraphic imaging, 1, 2 or if necessary 3 hours post injection, the excision of the primary tumour and the sentinel node procedure are both guided by a gamma probe. At the site of maximum counts with the gamma probe (Europrobe, Strassbourg, France) patent blue (Bleu patenté V 'Guerbet')is injected intratumourally (see Figure 1).

As with all localization techniques, care is required in the initial placement of the lesion marker. A few studies have described failures in placement of the radioactive marker. However, the tracer was positioned correctly in 95–99% of patients[6,7]. After localization, the surgical excision is guided by the probe at its lowest sensitivity setting. The exact site of the lesion can be checked constantly during the procedure by using the probe. In this way centering of the lesion within the specimen can be achieved, potentially resulting in a smaller quantity of removed tissue and a higher chance of achieving tumour free margins.

### WGL procedure

Patients in the WGL group will receive a guide wire, positioned intratumourally under ultrasonigraphic or stereotactic guidance *after *the scintigrafic imaging 1, 2 or 3 hours post injection. The excision of the tumour is guided by the inserted wire and the sentinel node procedure is performed using per operatively injected patent blue and a gamma probe. This is the current golden standard.

### Outcome parameters

The outcome parameters are tumour free margins (and number of re-excisions), resection specimen volume, cosmetic outcome, quality of life, cost effectiveness also participating physicians will score the difficulty of the procedures. Patients will be asked to fill in questionnaires on pain during the procedure, quality of life and the cosmetic outcome. A specific burden questionnaire, aimed at evaluating the burden of the cosmetic result, is developed. Such an instrument is currently not available. To further assess the net impact in terms of Health Related Quality of Life (HRQoL) also the EQ5D and the EQVAS will be obtained at T = 0, 6, 12 and 26 weeks after the initial diagnostic work-up.

The outcome of the study can be divided in 3 groups of outcome; negative outcomes, outcomes open to discussion and positive outcomes.

Less tumour free margins in the ROLL group are considered negative outcomes, also more tumour free margins in combination with a larger resection volume is a negative outcome and equal tumour free resection margins combined with larger resection specimens are considered negative. Secondly outcomes open to discussion are more tumour free margins in combination with an equal resection volume and equal resection margins in combination with smaller resection specimens in the ROLL group. Finally positive outcomes are more tumour free resection margins in combination with smaller resection specimens and equal tumour free margins in combination with smaller resection specimens. These data will be based on pathology reports. All surgical specimens will be analysed by one dedicated reference pathologist (PvD).

The overall outcome will be cost-effectiveness in terms of incremental costs per quality adjusted life years gained with a 6 month time horizon. We hypothesize that the short-term burden will result in an overall superior result after ROLL.

When indeed our expectations are evidenced, than a situation of dominance may occur, i.e., costs may be saved while at the same time clinical effects are optimal after ROLL.

Should the results indicate that overall the WGL procedure leads to better clinical outcome a cost-effectiveness analysis is foreseen using bootstrapping to assess the uncertainty with regard to the balance between costs and effects. All analyses will be limited to a half year time horizon. Accordingly, discounting of costs or effects is not applicable.

### Sample size

Sample size is calculated based on the primary endpoint: tumour free margins. Based on currently available literature we assume a difference in tumour free margins of 15% in favor of the ROLL procedure [8-14]. With a statistical power of 80% to detect this 15% improvement as significant (p < 0.05), we will require 158 patients in the control (WGL) group and 158 in the ROLL group.

Assuming a weight of 50 gr in the WGL group and a difference of 10 gr in the ROLL group [15] and a standard deviation of 3 gr, α = 0.05 and β = 0.80, the groups are sufficient to show a significant reduction in weight.

### Economic evaluation

The goal of the economic evaluation is to assess the balance between costs and effects of ROLL versus WGL. The principal underlying assumption is that once tumour free margins are obtained the prognosis will be similar.

Therefore, long term analyses will not be necessary. In fact ignoring the possible difference in burden associated with WGL as compared to ROLL a cost-minimization would suffice. This is the initial approach, i.e., comparing actual costs incurred with both strategies up until 6 months after the first operation. Costs estimates will be based on the actual costs of both procedures. This includes the costs of operation rooms, hospital stay, ultrasound/stereotactic imaging, wire placement, gamma probe use and if necessary, costs associated with complications and re-operations.

To assess the impact of the psychological burden of prolonged uncertainty regarding curative excision and the psychological burden associated with re-operation, dedicated questionnaires are developed.

Finally, there is a possibility that after ROLL, on average, a larger part of the breast is excised leading to less satisfactory cosmetic results. This is evaluated by interviewing the patients on their appreciation of the shape and appearance of their breast.

### Statistical analysis

The difference in radicality of the resected specimen in both groups will be calculated in a confidence interval (CI) of 95%.

The volume of the specimens is presented in mm^3 ^and the maximum diameter in mm. The approach for the cost-analysis is comparing actual costs incurred with both strategies up until 6 months after the first operation. Costs estimates will be based on the actual costs of both procedures. This includes the costs of operation rooms, hospital stay, ultrasound/stereotactic imaging, wire placement, gamma probe use and if necessary, costs associated with complications and re-operations.

The degree of difficulty of radiological and surgical procedure will be expressed on a 1–10 scale (1 being extremely easy and 10 extremely difficult). The average score for both procedures will be calculated. Patient discomfort of the radiological procedure is expressed on 1–10 scale to (1 being not painful and 10 being very painful).

The success rate of the sentinel node procedure is presented in a percentage of successfully detected and found sentinel nodes.

The weight and size of the surgical specimens as well as the degree of difficulty of the surgical and radiological procedures and patients discomfort in both groups will be analyzed using the students T test.

The cost-effectiveness analysis will be done using the multivariate analysis. If the baseline characteristics differ after randomization, i.e. there is a lack of balance in the confounding factors, this will be corrected using the multivariate analysis.

## Discussion

The purpose of the ROLL trial is to compare the standard of care (WGL) with the ROLL procedure on oncological outcome, cost effectiveness, cosmetic outcome, patient comfort and learning curve for the participating physicians.

As published previously by our group, the published studies so far indicate a benefit of the ROLL procedure, but are not conclusive [5]. So far only 1 randomized clinical trial is available, but this trial lacks essential information on tumour free margins and tumour size [16].

A large trial on ROLL published recently including 368 patients showed tumour free margins in 89% of the cases and a 97% identification rate of the sentinel node [17]. These figures are encouraging, but the patients in this study were not randomized, and a hook wire was always inserted at the primary tumour site. In our opinion this will not improve patient comfort and the technique is not easier to perform.

In some studies, patients with non palpable invasive carcinoma, carcinoma in situ and benign lesions were included [18,19]. However, patients with an invasive carcinoma are likely to benefit the most from the ROLL technique. As these patients have to undergo a SNB, they will have to undergo one procedure less, i.e. the placement of a wire.

To the best of our knowledge none of the studies published have evaluated cost-effectiveness. In order to implement the new procedure nationwide, besides oncological outcome the cost-effectiveness should be balanced to make this the new standard of care. Approximately 4.000 out of 13.000 women diagnosed with breast cancer need wire guided localization and a sentinel node procedure. Our hypothesis, based on the current literature, is that 15% (600 patients) less re-operations are necessary after treatment with the ROLL technique. A re-operation costs about € 7000, so eventually € 4.200.000 a year might be saved by this method. Furthermore, abandoning the radiological wire insertion procedure and the shorter surgical operating times could provide an additional cost reduction.

In conclusion, the ROLL trial aims to prove oncological, patient satisfaction and cost effective superiority of the ROLL technique versus the WGL technique in the treatment of patients with a non palpable breast carcinoma.

## Abbreviations

ROLL: Radio guided occult lesion localization; WGL: Wire guided localization; SNB: Sentinel node biopsy; Gr: Grams; HRQOL: Health related quality of life; EQ5D: EuroQuol 5d; EQVAS: EuroQuol visual analogue scale.

## Authors' contributions

SvE drafted the manuscript. IMvdP co-authored the manuscript. SvE, MGGH, PHMP, EB, IMvdP, WPThM, IHMBR and RvH participated in the design of the protocol. IMvdP, PHMP and EB performed the sample size calculations. All authors edited the manuscript and approved the final manuscript.

## Pre-publication history

The pre-publication history for this paper can be accessed here:


